# Half-metallicity and ferromagnetism in *penta*-AlN_2_ nanostructure

**DOI:** 10.1038/srep33060

**Published:** 2016-09-12

**Authors:** Jiao Li, Xinyu Fan, Yanpei Wei, Haiying Liu, Shujuan Li, Peng Zhao, Gang Chen

**Affiliations:** 1 Laboratory of Advanced Materials Physics and Nanodevices, School of Physics and Technology, University of Jinan, Jinan, Shandong 250022, China

## Abstract

We have performed a detailed first-principles study of the *penta*-AlN_2_ nanostructure in the Cairo pentagonal tiling geometry, which is dynamically stable due to the absence of imaginary mode in the calculated phonon spectrum. The formation energy and the fragment cohesive energy analyses, the molecular dynamics simulations, and the mechanical property studies also support the structural stability. It could withstand the temperature as high as 1400 K and sustain the strain up to 16.1% against structural collapse. The slightly buckled *penta*-AlN_2_ is found to be a ferromagnetic semiconductor. The strain of ~9% could drive the structural transition from the buckled to the planar. Interestingly, the strain of >7% would change the conducting properties to show half-metallic characters. Furthermore, it could be also used to continuously enhance the magnetic coupling strength, rendering *penta*-AlN_2_ as a robust ferromagnetic material. These studies shed light on the possibilities in synthesizing *penta*-AlN_2_ and present many unique properties, which are worth of further studying on both theory and experiment.

As a wide band gap semiconductor, the aluminum nitride (AlN) with a band gap of 6.2 eV has been characterized to have strong bonds being of ~20% shorter than those of the other III-V semiconductors and high ionicity being of roughly two times higher than the data of the other III-V materials, which also has good dielectric properties, high thermal conductivity, and low thermal expansion coefficient. These superior properties make it as an attractive material for ultraviolet optoelectronics and spintronics^1^,^2^,^3^,^4^. The AlN bulk crystallizes in the wurtzite structure in P63mc space group with two interpenetrating hexagonal lattices indicating the possibility for forming the hexagonal network nanostructures. The AlN nanostructures existing in the form of nanocones, nanotubes, nanoribbons, nanosheets, and nanowires have attracted considerable research attention on both experiment^5^,^6^,^7^,^8^,^9^,^10^,^11^,^12^,^13^,^14^ and theory^15^,^16^,^17^,^18^,^19^,^20^,^21^,^22^,^23^,^24^. In nanostructures, the AlN hexagon is the predominating structural unit. Similar to the carbon, the AlN could also form the two-dimensional (2D) honeycomb nanostructure *h*-AlN, which is the base material for the AlN nanocone, nanotube, and nanoribbon. Attributed to the quantum effects, the nanostructures are found to have many unique properties. For example, Wang *et al*. carefully studied the hydrogen storage of the AlN nanocage, nanocone and nanotube. In comparison with the AlN bulk with the four-fold coordinate Al, these nanostructures attributed to the lower coordination of Al provide desirable adsorption sites to bind H_2_ molecules in quasi-molecular form through the charge polarization mechanism, leading to the 4.7 wt% hydrogen storage ability^15^. Their attractive electronic, magnetic, and optical properties have also been extensively studied^3^,^5^,^6^,^7^,^8^,^9^,^16^,^17^,^18^,^19^,^20^,^21^,^22^,^23^,^24^. Qi *et al*. showed the modification of the electronic structure of AlN nanoribbons by doping C, in which the band gap could be continuously tuned^20^. Du *et al*. found the spin-anisotropic transport properties of the zigzag AlN nanoribbons with N edge unpassivated. The H-termination would quench the magnetic properties and make the zigzag nanoribbons to be indirect band gap semiconductors^21^. Cao *et al*. studied the transition metal doped AlN nanosheets, in which the double exchange mechanism mediated ferromagnetic (FM) coupling and the magnetic interaction tuning by the in-plane tensile strain were carefully investigated^24^.

It is a long-term standing challenge to explore the advanced materials by modifying or changing material’s structure, addressing the fundamental research issue of structure-property in the field of materials physics. The honeycomb network geometry entirely composed of hexagons is well known for most of the currently studied 2D materials, including the graphene, its same column cousin materials (silicene, germanene, and stanene), and lots of inorganic nanosheets (such as the *h*-BN, *h*-AlN, MoS_2_). Very interesting, a most recent study performed by Zhang *et al*. confirmed the pure pentagon-based 2D nanomaterial *penta*-graphene resembling the Cairo pentagonal tiling in geometry, which corresponds to the layer structure of T12-carbon bulk^25^. Wang and coworker also studied its thermal conductivity^26^ and proposed a method for tuning its electronic and mechanical properties^27^. Inspired by this finding, the hydrogenated silicene^28^, B_2_C^29^, BN_2_^30^, and CN_2_^31^ in pentagonal lattices have also been recently reported. As a contribution to the structure-property issue, we have carried out a detailed investigation on the aluminum nitride nanomaterials with the Cairo pentagonal tiling geometry. The pentagonal AlN_2_ is found to be stable confirmed by our formation energy and fragment cohesive energy studies, the dynamic stability analyzed by the phonon spectrum, the stability against temperature suggested by our study of melting temperature, and the mechanical stabilities against tensile strain. The structural transition as well as the transition from semiconducting to half-metallic could occur at the aid of strain loading. The ground state of the pentagonal AlN_2_ is the FM coupled magnetic state, which also shows robust magnetic properties against the tensile strain. These studies could shed light on the possibilities in synthesizing pentagonal AlN_2_ and present many unique properties, which would be interesting to call for further studies on both theory and experiment.

## Results

### Geometrical structure and dynamic stability

**Figure 1:**
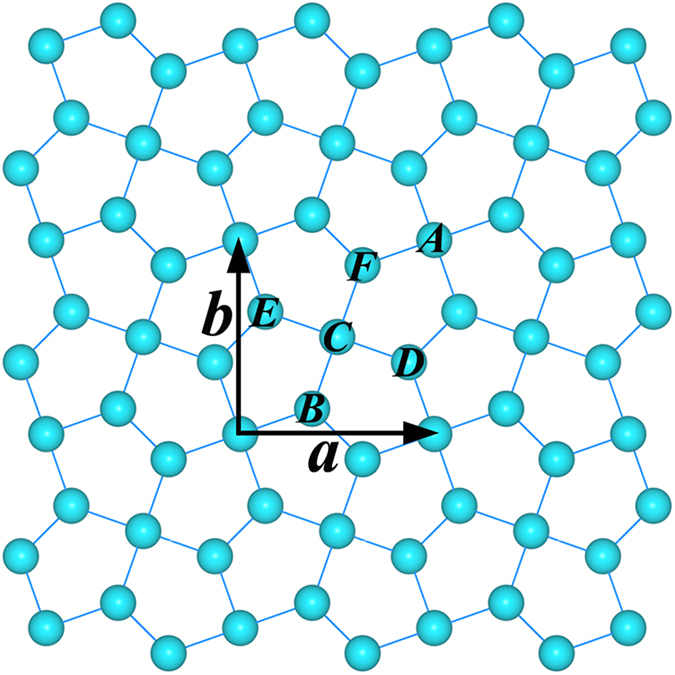
The schematic illustration of the Cairo pentagonal tiling geometry with the a* *× b repeated unit highlighted.

**Figure 2:**
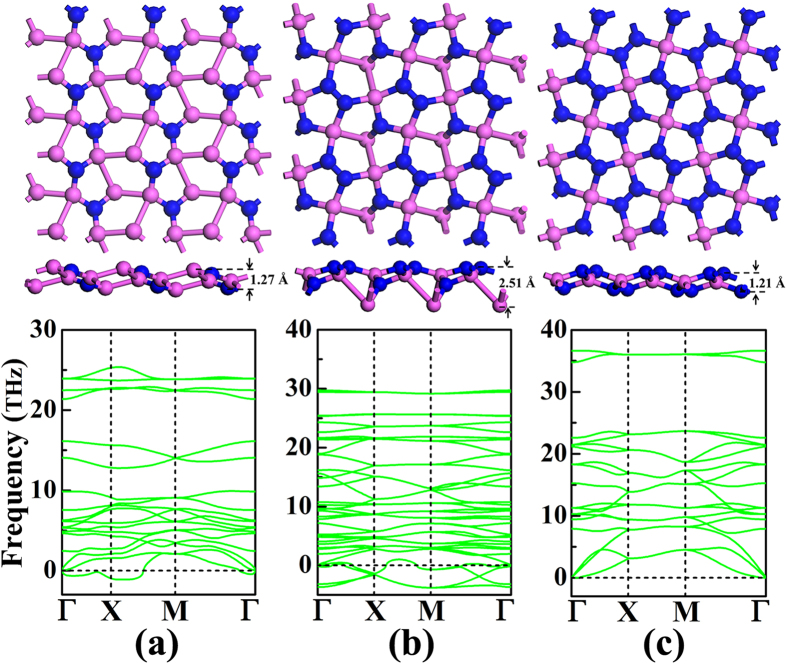
The optimized geometrical structures of pentagonal sheet and the corresponding phonon spectra for Al_2_N (**a**), AlN (**b**), and AlN_2_ (**c**). The purple and blue balls are for the Al and N atoms, respectively.

The Cairo pentagonal tiling geometry has been shown in Fig. 1, in which the repeated unit is highlighted by the ***a*** × ***b***. Considering all the non-equivalent configurations, we have carefully carried out full optimizations of the geometrical structures and the lattice constants. As shown in Fig. 2, only three sheet structures for the Al_2_N, AlN, and AlN_2_ nanomaterials are found to remain, which are slightly buckled as quasi-planar structures to release the local steric strain. The displacement distances between the top atomic layer and the bottom layer are measured to be 1.27, 2.51, and 1.21 Å for the Al_2_N, AlN, and AlN_2_ nanomaterials, respectively. The atomic nitrogen species are separately distributed in the aluminum network in the Al_2_N. The AlN consists of atomic nitrogen, dinitrogen, and Al_3_ chains. As in the AlN_2_, there are only atomic aluminum and dinitrogen composition species. With the purpose to evaluate their dynamic stabilities, we have then performed phonon dispersion analyses, which are presented in Fig. 2. It is clear that the Al_2_N and AlN in pentagonal lattices are unstable due to the presence of imaginary modes in the calculated phonon spectra. The structure in Cairo pentagonal tiling geometry could only stand for the AlN_2_, referred as *penta*-AlN_2_ hereafter to facilitate discussion. Here, we would like also to mention the experimentally fabricated Ti_8_C_12_ metallo-carbohedrene which is composed of Ti atoms and C_2_ dimers^32^. Similarly, our previous studies of the calcium metal carbides also support the C_2_ dimers as preferable composition fragments^33^,^34^. The composition in the form of dinitrogen could also be seen in the recently reported carbon nitride materials^31^. Actually, the fabrication method of compressing metal materials with the source of N_2_ molecules at high pressure^35^,^36^ was previously applied to successfully synthesize transition metal nitrides composed of metal and dinitrogens. All of these studies shed light on the possibilities in synthesizing the dinitrogen composed *penta*-AlN_2_ nanostructure.

### Energetic stability

For the *penta*-AlN_2_, we have calculated the formation energy. The primitive unit cell is used in the calculation, which contains 2 Al atoms and 2 N_di_ dinitrogens.


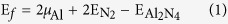


where the *μ*_Al_, 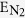, and 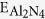 stand for the chemical potential of aluminum metal, the total energy of N_2_ gas molecule, and the total energy of the *penta*-AlN_2_ calculated for the primitive unit cell. Then, the formation energy is found to be 0.65 eV per primitive unit cell, showing exothermic properties in fabricating *penta*-AlN_2_. By using a supercell of 5 × 5 × 1 to minimize the structural deformation effects from the neighboring images of a vacancy defect, we have also calculated the fragment cohesive energies of the composition species by the below formula.


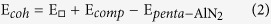


where the E_*comp*_ stands for the energy calculated for a free-standing N_2_ molecule or the chemical potential of aluminum metal. The 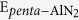 and E_□_ are the total energies calculated for the *penta*-AlN_2_ nanostructure and the vacancy defected *penta*-AlN_2_ structure optimized after removing the composition species. The fragment cohesive energies for a Al atom and a N_di_ nitrogen are 5.07 and 2.98 eV, suggesting the exothermic reaction characters also.

### Thermal stability

**Figure 3:**
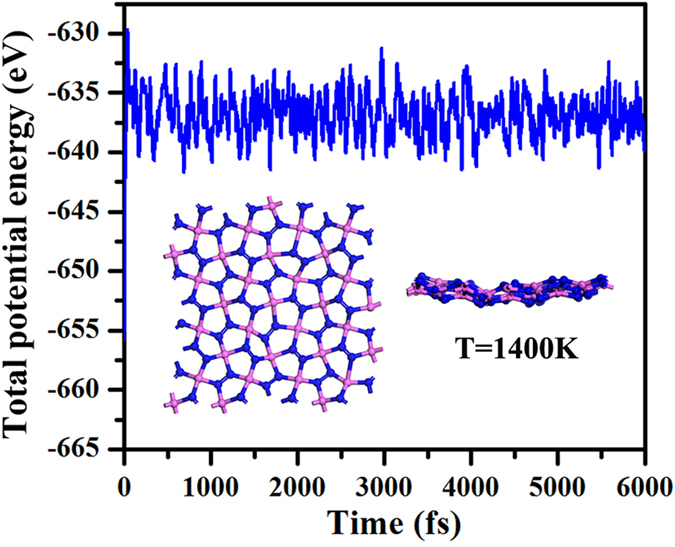
The molecular dynamics simulation for the *penta*-AlN_2_ nanostructure at the highest temperature that it could withstand against melting. The insets are the top and side views of the geometrical structure obtained at the end of the simulation. The purple and blue balls are for the Al and N atoms, respectively.

In our studies, the melting temperature of the *penta*-AlN_2_ has been evaluated using the *ab initio* molecular dynamics (AIMD) simulations to estimate the thermal stability. The AIMD simulations have been performed starting from the temperature of 300 K. Due to the intensive computing loading of AIMD simulations, the melting temperature is only estimated with the precision of 50 K. In our studies, the *penta*-AlN_2_ has been found to withstand the temperature as high as 1400 K. The Fig. 3 shows the evolution of the total potential energy as a function of the simulation time for the AIMD simulation at 1400 K. The snapshot of the structure at the end of the simulation has also been shown. The structural skeleton remains though the structural distortion happens. Taking the structure obtained at the end of the simulation as starting configuration, we have then fully relaxed the structure which would quickly converge to the quasi-planar *penta*-AlN_2_ structure by releasing 1.25 eV energy, confirming the thermal stability.

### Mechanical stability

**Figure 4:**
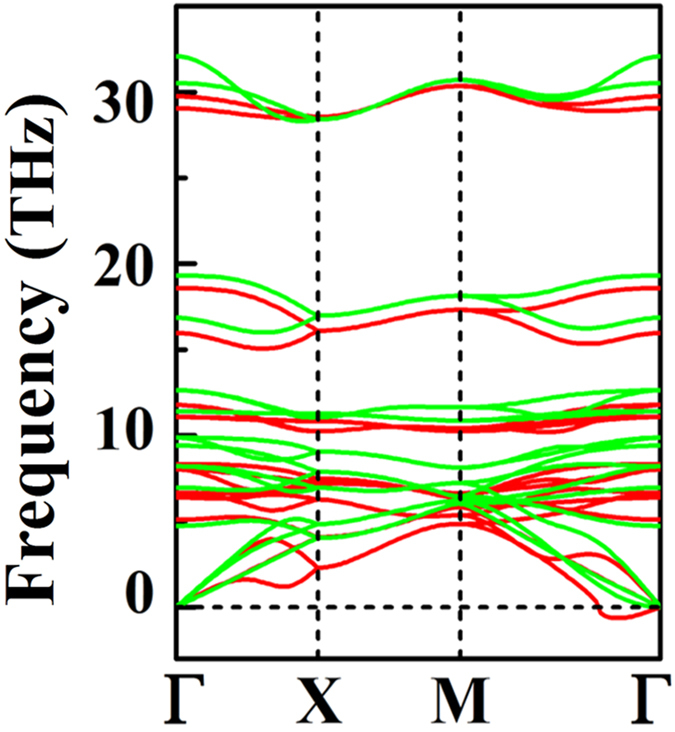
The phonon spectrum for the *penta*-AlN_2_ at the extreme of the applied tensile strain before structural collapse. The green and red lines are for the spectra calculated for *penta*-AlN_2_ under 16.1% and 16.2% strains, respectively.

Considering the application usage, the mechanical stability of the *penta*-AlN_2_ also needs to be estimated. After applying strain, the geometrical structure was fully relaxed and its phonon spectrum was then studied. In Fig. 4, the calculated phonon spectrum for the *penta*-AlN_2_ under 16.2% tensile strain start to show imaginary modes, suggesting its stability to withstand stain of as high as 16.1%. Besides, by using the finite distortion method^25^, the linear elastic constants of the *penta*-AlN_2_ have been calculated. The elastic modulus tensors *C*_11_, *C*_22_, *C*_12_, and *C*_66_ can be obtained by analyzing the second partial derivative of strain energy with respect to strain, which are calculated to be 115.73, 115.73, 1.93, and 51.62 N/m for *penta*-AlN_2_. They satisfy the conditions of 
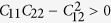
 and 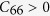 to support the mechanical stability of *penta*-AlN_2_ according to the Born-Huang criteria^37^. Also, based on the obtained elastic constants, we have estimated the in-plane Young’s moduli for the *penta*-AlN_2_ and the graphene-like hexagonal *h*-AlN. The calculated value of 115.70 for *penta*-AlN_2_ is close to the 123.52 of *h*-AlN, showing comparable stiffness.

## Discussion

### Electronic bandstructures

**Figure 5:**
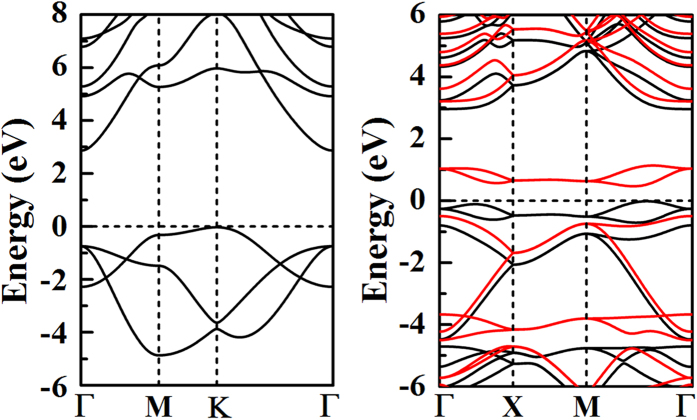
The electronic bandstructures for the stable hexagonal *h*-AlN sheet (left) and the stable pentagonal *penta*-AlN_2_ sheet (right). In the right panel, the black and red lines are for the spin-up and spin-down electrons, respectively.

The attractive properties of low-dimensional nanostructures of AlN have aroused extensive research attention on both theory and experiment. The commonly studied 2D material is the graphene-like *h*-AlN, in which the intersected Al-sublattice and N-sublattice form the hexagonal honeycomb structure. Different with the wurtzite bulk material, the *h*-AlN turns to be indirect band gap semiconductor, addressing the fundamental issue of structure-property relation in the field of material physics. As for the *penta*-AlN_2_ sheet, the structure fully composed of pentagons in the Cairo pentagonal tiling geometry may also impose novel properties attributed to the unique topology and reduced dimensionality. The calculated electronic bandstructures for the hexagonal *h*-AlN and pentagonal *penta*-AlN_2_ are presented in Fig. 5. In comparison with the *h*-AlN, the *penta*-AlN_2_ turns to be magnetic semiconductor. Both spin-up and spin-down electrons have indirect band gaps, which are 3.0 and 0.9 eV, respectively. The band structures of *penta*-AlN_2_ show typical characters of bipolar magnetic semiconductors (BMS). The idea of the BMS was firstly proposed by Yang and coworkers in studying semi-hydrogenated single-walled carbon nanotubes^38^. By controlling gate voltage, one could realize the electric control of spins in *penta*-AlN_2_, rendering it attractive spintronics material. All of the bands along the XM path show double degeneracy protected by the P-42_1_m structural symmetry, which is the universal band degeneracy characters of the Cairo pentagonal tiling nanostructures^25^,^26^,^27^,^28^,^29^,^30^,^31^. The charge distributions of the valence and conduction bands are found to be located around N atoms. Like the *penta*-CN_2_ nanomaterial^31^, the interaction between Al and N_di_ in the *penta*-AlN_2_ induces the band splitting. The interatomic distance is reported to be 1.45 Å of the N_di_ in *penta*-CN_2_ to show singly bonding characters, making it to be high energy density material. The N_di_ in *penta*-AlN_2_ has bond length of ~1.37 Å between the 1.10 Å of triple-bond N ≡ N and the 1.45 Å of single-bond N–N, indicating the double-bond N = N characters.

### Bonding nature in *penta*-AlN_2_

**Figure 6:**
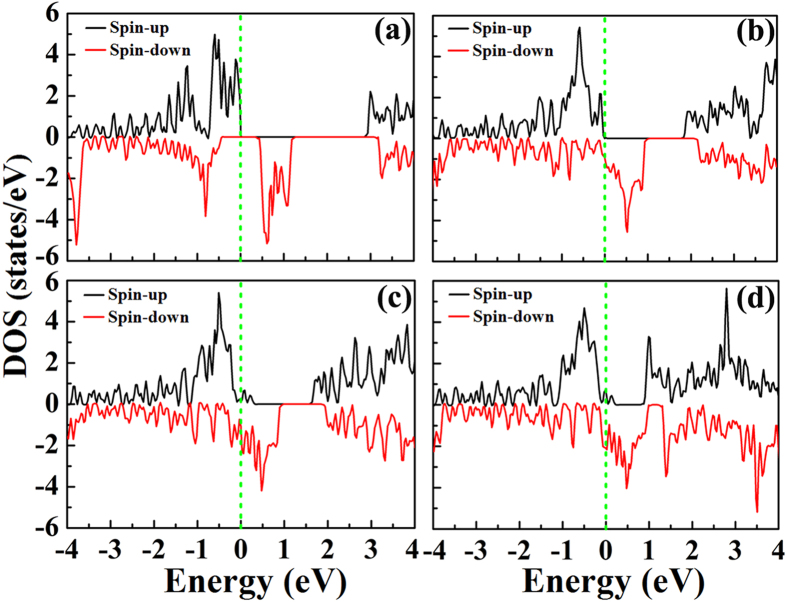
The density of states for the free-standing *penta*-AlN_2_ (**a**). The (**b**–**d**) are for the corresponding densities under strains of 7% (half-metallic properties start to appear), 9% (structural transition from the buckled to the planar), and 16% (extreme of strain before structural collapse).

**Figure 7:**
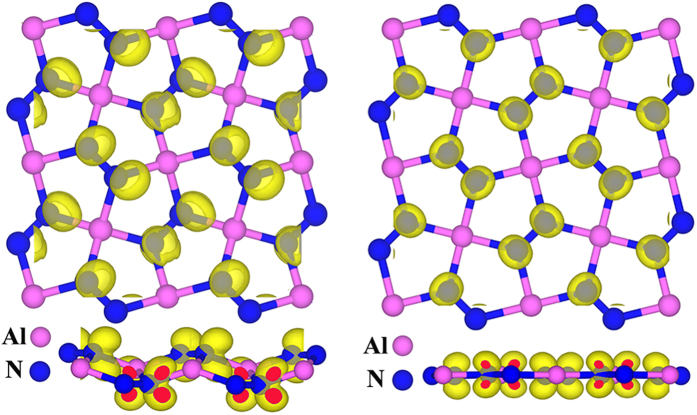
The illustration of the magnetism distributions before and after structural transition. The left and the right panels are for the free-standing *penta*-AlN_2_ and the one under 9% strain. The iso-surfaces at 0.008 *μ*_B_/Å^3^ are plotted.

The electronic bandstructure in Fig. 5 and the DOS in Fig. 6 suggest the *penta*-AlN_2_ to be magnetic. The physical origin of the magnetic properties lies in the band splitting induced by the interaction between Al and N_di_, which results in the valence and conduction bands. The calculated magnetic moment is 1 *μ*_B_ per primitive unit cell. In Fig. 7, the magnetism distributions are presented for the free-standing *penta*-AlN_2_ with the buckled sheet structure and the one under 9% tensile strain in the ideal planar geometry, which show the N atoms to be magnetic. In our studies, strain would not change the fact of the magnetism distributions on N atoms. In comparison with the N ≡ N configuration of nitrogen molecule, the dinitrogen N_di_ in the *penta*-AlN_2_ develops double bonds N = N, which would also bond with the nearest Al atoms to form the 3-coordinate N and 4-coordinate Al atoms. After isolating a N = N from the *penta*-AlN_2_ and freezing its atomic positions, we have calculated the total energies in both neutral and negatively charged states to estimate the electronic affinity. The calculated electronic affinity is ~1.95 eV, making N = N tend to attract the electrons of Al. We would like to mention the electronegativity of Al atom is 1.61. The sum of the Al and N atomic radii are 1.92 Å and the Al-N interatomic distance in free-standing *penta*-AlN_2_ is 1.90 Å, indicating covalent-like bonding. The covalent-like bonding characters were also previously reported in Al nanostructures^39^,^40^,^41^. The bonding characters in nanostructures could be analyzed by using the electron localization function (ELF) analysis^39^,^40^,^41^,^42^,^43^,^44^, which was introduced in quantum chemistry to measure the parallel spin correlation by defining conditional probability of finding an electron in the neighborhood of another electron with the same spin. The ELF is defined as


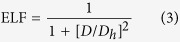



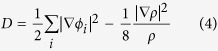



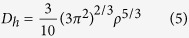


where 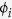 is the Kohn-Sham orbital, and *ρ* is the local density. Our calculated ELF data of 0.8 between Al and N confirms the weak covalent-like nature of Al-N bonds of the *penta*-AlN_2._

### Strain induced structural transition and half-metallicity

Strain engineering is believed to be effective in modulating electronic properties of the two-dimensional materials^29^,^45^,^46^. Li *et al*. found that the buckled structure of *penta*-B_2_C could be stretched to be planar under 15% tensile tensile strain. Along with the structural transition, the band gap could be strikingly reduced from 2.28 eV to 0.06 eV. Certain strain can drive the *penta*-B_2_C to be metallic^29^. For our studied *penta*-AlN_2_, the strain can also affect its transport properties, making it to show half-metallic conducting characters. After applying tensile strain, we have carefully investigated the geometrical structure and electronic properties of *penta*-AlN_2_. For the *penta*-AlN_2_, the strain of 9% would realize the structural transition from the buckled quasi-planar to the ideal planar. The strain would continuously elongate the Al-N interatomic distance while it would not affect the N-N interatomic distance. For example, the data are calculated to be 1.90 and 1.37 Å for Al-N and N-N of the free-standing *penta*-AlN_2_, which are 2.14 and 1.37 Å calculated under the 16% strain, respectively. The strain induced bond length increase of Al-N bond hints that the corresponding interaction would be weakened. At the structural transition point of 9% tensile strain, the Al-N length is found to be 2.0 Å, which has been elongated by 5.3%. Besides the structural transition, the conducting properties of the *penta*-AlN_2_ would also bear the effects of strain. In Fig. 6, the densities of states of the *penta*-AlN_2_ with and without strains have been shown. The free-standing material is semiconducting. The spin-up and spin-down densities show 3.0 and 0.9 eV band gaps. When the applied tensile strain is increased to 7%, the *penta*-AlN_2_ would change to half-metallic. The energy gap of spin-up electrons is 1.8 eV while the spin-down electrons could transport freely. In comparison with the N ≡ N in N_2_ gas molecule, the interaction between Al and N accounts for the breaking of one of the bonds to get the N = N doubly bonding in N_di_ in *penta*-AlN_2_, which forms valence and conduction bands. By applying strain, the distance between Al and N would continuously enlarged, which in turn would weaken their interaction to affect the binding energies of the valence and conduction bands. Under 7% tensile strain as shown in Fig. 6b, the band gap of spin-down electrons would be closed, while the spin-up band gap still remains opening. Further enhancing the strain, it would be changed to metallic for both spin-up and spin-down electrons. Figure 6c shows the calculated DOS for the *penta*-AlN_2_ under the tensile strain of 9% which makes the structural transition from the buckled to the planar. The DOS calculated under the tensile strain as large as 16% also shows metallic properties. Carefully analyzing our calculated DOS, we would like to say that the DOSes for the *penta*-AlN_2_ under strain in the range from 7% to the maximum 16.1% before structural collapse show overall analogous characters in the proximity of Fermi level. As illustrated in Fig. 6c,d, the densities in the range of ~1 eV above Fermi level are obvious for the spin-down electrons while they are almost negligible for the spin-up electrons, indicating the transport properties to be half-metallic like.

### Room-temperature ferromagnetism

**Figure 8:**
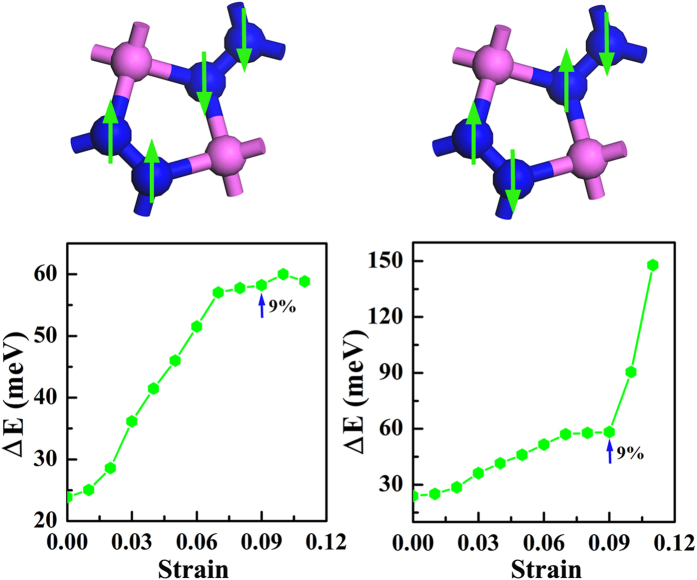
The evolution of the magnetic coupling strength (the lower) versus tensile strain and its corresponding AFM configuration (the upper). The arrow marks the point where the structural transition from the buckled to the planar geometries happens.

The ground state configuration of the magnetic coupling is ferromagnetic. Two configurations for the antiferromagnetic (AFM) coupling are considered in our studies. As shown in Fig. 8, they are the AFM coupling among the neighboring N_di_ while the N atoms in N_di_ keep FM coupling and the other coupling configuratioin for N atoms in N_di_ to have contrary spin polarizations. Hereafter, in order to facilitate discussion, we would like to refer them as N_di_-AFM and N-AFM, respectively. In the free-standing *penta*-AlN_2_, they are almost degenerate in energy. The coupling strength could be estimated by the energy difference as defined below:


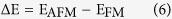


where E_AFM_ and E_FM_ are the calculated total energies for the AFM and FM coupling states, respectively. The ΔE is calculated to be ~24 meV for the free-standing *penta*-AlN_2_. According to the mean field approximation (MFA), the Curie temperature (T_c_) is estimated to be ~371 K^47^,^48^,^49^. Furthermore, studies of the strain effects presented in Fig. 8 show robust magnetic properties of *penta*-AlN_2_. Before structural transition, the coupling strength could be continuously enhanced in the buckled structure. Once the structure transition happens, the N-AFM in the planar structure would be largely suppressed. The configuration of two N atoms in N_di_ having same polarization bears the effects of the bond breaking when comparing the N = N with the inert N ≡ N species. Once the N ≡ N forms, the *penta*-AlN_2_ would be destroyed. Our mechanical stability studies show that the structure could sustain the strain loading up to 16.1%. So, in the case of the strain loading such as the 9% strain, the *penta*-AlN_2_ is the stable structure to keep the N = N configuration. In the free standing *penta*-AlN_2_, the Al-N bonding is strong enough, and the spin-up and spin-down electronic bands are actually well separated in binding energy. However when the strain loading increases up to >9%, slight overlap between spin-up and spin-down electrons starts to occur (for example, see Fig. 7c). Then, the unparallel polarized electrons would have the chance to fill in the degenerate states to harm the N = N configuration, which is unfavorable for keeping the *penta*-AlN_2_ structure under the tensile strain in the range from 9% to 16.1%. Therefore, the N_di_-AFM becomes the preferred AFM coupling state for planar structure. Also, the coupling strength could be further enhanced. Though the MFA may overestimate the T_c_, our calculated data are an indication that the *penta*-AlN_2_ could keep magnetic moment at room temperature at the aid of strain. Also, probably due to the covalent-like bonding nature between Al and N, the calculated magnetic moment keeps almost unchanged against strain loading in our studies.

In summary, we have carried out detailed first-principles investigations of the *penta*-AlN_2_ nanostructure composed of pentagons in the Cairo pentagonal tiling geometry, whose stability has been carefully evaluated. The calculated phonon spectrum confirms its dynamic stability due to the absence of imaginary mode. The melting temperature is estimated to be >1400 K by our AIMD simulations to show high thermal stability against the temperature. The formation energy and the fragment cohesive energy analyses also support the structural stability. According to our mechanical property study, the *penta*-AlN_2_ could sustain the strain up to ~16% against structural collapse. Attributed to the unique topology and reduced dimensionality, the *penta*-AlN_2_ is found to be an indirect band gap ferromagnetic semiconductor. The ground state is the slightly buckled quasi-planar sheet structure to release the local steric strain, resulting in the atomic layer of 4-coordinate Al atoms sandwiched between the top and bottom layers of dinitrogens. The structural transition from the buckled to the planar would occur under the strain of 9%. Interestingly, by applying strain of >7%, the *penta*-AlN_2_ could be used as a half-metallic material. Furthermore, the strain could also be used to continuously enhance the magnetic coupling strength, rending *penta*-AlN_2_ as a robust ferromagnetic material. These studies shed light on the possibilities in synthesizing *penta*-AlN_2_ and present many unique properties, which are worth of further studying on both theory and experiment.

## Methods

All of the calculations were carried out by using the Vienna *ab initio* Simulation Package (VASP)^50^ code based on the density functional theory (DFT). The generalized gradient approximation with Perdew, Burke, and Ernzerhof (PBE)^51^ parameterization was used to describe the exchange-correction interaction among electrons, and the projector augmented wave psedopotential (PAW)^52^ method was adopted to deal with the ion-electron interaction. The cutoff energy for the plane-wave basis set was chosen to be 400 eV. We have put the sheet structure in the *xy* plane with its perpendicular direction as *z* axis, and a vacuum space of of 15 Å in *z* direction was adopt to minimize the interaction between the sheet structure and its neighboring periodic images. The Monkhorst-Pack scheme^53^ of a 15 × 15 × 1 Γ -centered *k* point mesh was applied for the structural optimizations and electronic structure studies. All the atoms were fully relaxed with the energy and force convergences up to 10^−5^ eV and 0.02 eV/Å, respectively. The *ab initio* molecular dynamics simulations were performed by using the canonical ensemble to evaluate the thermal stability. The supercell of 4 × 4 × 1 was adopted to release the periodic constraints on atomic spacial movement. Each simulation lasted for 6 ps with the time step of 1 fs. The dynamic stability of the nanostructure was checked by the phonon dispersion analysis using the Phonopy code with the finite displacement method^54^, which is interfaced with the density functional perturbation theory as implemented in VASP. For calculating the phonon spectrum, the energy convergence criteria were set to 10^−8^ eV for the total energy and 0.1 meV/Å for the calculated Hellmann-Feynman force.

## Additional Information

**How to cite this article**: Li, J. *et al*. Half-metallicity and ferromagnetism in *penta*-AlN_2_ nanostructure. *Sci. Rep.* **6**, 33060; doi: 10.1038/srep33060 (2016).
